# Inappropriateness of Cardiovascular Radiological Imaging Testing; A Tertiary Care Referral Center Study

**DOI:** 10.1371/journal.pone.0081161

**Published:** 2013-11-27

**Authors:** Clara Carpeggiani, Paolo Marraccini, Maria Aurora Morales, Renato Prediletto, Patrizia Landi, Eugenio Picano

**Affiliations:** 1 CNR, Institute of Clinical Physiology, Pisa, Italy; 2 Fondazione CNR-Regione Toscana Gabriele Monasterio, Pisa, Italy; University of York, United Kingdom

## Abstract

**Aims:**

Radiological inappropriateness in medical imaging leads to loss of resources and accumulation of avoidable population cancer risk. Aim of the study was to audit the appropriateness rate of different cardiac radiological examinations.

**Methods and Principal Findings:**

With a retrospective, observational study we reviewed clinical records of 818 consecutive patients (67±12 years, 75% males) admitted from January 1-May 31, 2010 to the National Research Council – Tuscany Region Gabriele Monasterio Foundation cardiology division. A total of 940 procedures were audited: 250 chest x-rays (CXR); 240 coronary computed tomographies (CCT); 250 coronary angiographies (CA); 200 percutaneous coronary interventions (PCI). For each test, indications were rated on the basis of guidelines class of recommendation and level of evidence: definitely appropriate (A, including class I, appropriate, and class IIa, probably appropriate), uncertain (U, class IIb, probably inappropriate), or inappropriate (I, class III, definitely inappropriate). Appropriateness was suboptimal for all tests: CXR (A = 48%, U = 10%, I = 42%); CCT (A = 58%, U = 24%, I = 18%); CA (A = 45%, U = 25%, I = 30%); PCI (A = 63%, U = 15%, I = 22%). Top reasons for inappropriateness were: routine on hospital admission (70% of inappropriate CXR); first line application in asymptomatic low-risk patients (42% of CCT) or in patients with unchanged clinical status post-revascularization (20% of CA); PCI in patients either asymptomatic or with miscellaneous symptoms and without inducible ischemia on non-invasive testing (36% of inappropriate PCI).

**Conclusion and Significance:**

Public healthcare system – with universal access paid for with public money – is haemorrhaging significant resources and accumulating avoidable long-term cancer risk with inappropriate cardiovascular imaging prevention.

## Introduction

How the USA (and Europe) will pay for healthcare is a subject on the mind of virtually every American (and European) today. Are there areas where expenses can be cut without undermining the quality of care provided? One of these areas is certainly the inappropriate misuse and overuse of medical imaging [1]. The proliferation of cardiac imaging may represent added value when appropriate, and added cost when inappropriate [2]. The rate of inappropriateness was around 30% for stress echocardiography in our own lab [3], and similar values were found in laboratories of undisputed reputation in Australia, USA and South America for stress echo [3], [4], MPI [5], cardiac CT [6], CA [7] and even for PCI [8]. Appropriateness in healthcare is a moving target and not easy to define. Appropriateness criteria change rapidly as new evidence appears [9]; also, the cultural and economic climate has recently changed abruptly, with efforts by scientific societies to promote the culture of appropriateness [10]–[13] and of political governance to monitor inappropriateness in order to slow the fiscal bleeding [14], [15]. Inappropriateness in medical imaging is not only exorbitantly costly [2], but is also an avoidable risk for the patient when performed with ionizing radiation (in radiology and nuclear medicine), in spite of recent efforts by scientific societies [12], [13] and government [14], [15] to promote radiological responsibility and imaging appropriateness. Still, many medical acts (imaging, therapies, interventions) contribute to wasted money and decreased levels of safety in contemporary medicine [16], [17].

The aim of this study was to audit the level of appropriateness of four index cardiac radiology procedures, in a high-volume, tertiary care cardiovascular referral center in Tuscany, Italy. The monitored examinations reflected a wide spectrum of complexity, cost and radiation dose: chest x-ray (CXR), coronary computed tomography (CCT), diagnostic invasive coronary angiography (CA), coronary percutaneous interventions (PCI).

## Methods

### Ethics Statement

Written consent was given by the patients before an imaging procedure. The study was approved by the Pisa Ethical Committee as a part (work package 1) of the SUIT-Heart (Stop Useless Ionizing Testing in Heart Disease) study on October 1, 2010 (Study Protocol n.3005/2010).

### Study population

The CNR Institute of Clinical Physiology maintains an electronic database with data on all patients undergoing imaging procedures. With a retrospective, observational study design, we reviewed 940 consecutive medical imaging examinations performed during the period January 1-May 31, 2010 in Pisa, IFC CNR-RT FGM, in 818 patients: 250 CXR's (in 233 patients); 240 CCT's (in 240 patients); 250 CA's (in 245 patients); 200 PCI's (in 200 patients). For each examination, an independent expert clinical cardiologist (Head of the Cardiovascular Division, but not directly involved in the care of the audited patients) reviewed all available clinical records and scored the individual exam as: appropriate (class I definitely appropriate and class IIa, probably appropriate); uncertain (class IIb, probably inappropriate); definitely inappropriate (class III). Reference guidelines were those adopted in our Institute following the recommendations of the European Society of Cardiology [18] and American College of Cardiology/American Heart Association [19], [20]. Pre-test likelihood of coronary artery disease (CAD) by age, gender and symptoms for symptomatic patients and Framingham risk criteria to determine the risk of CAD for asymptomatic patients were calculated according to the European and ACC/AHA Guidelines for Chronic Stable Angina [18], [21]. Grading of angina pectoris by the Canadian Cardiovascular Society Classification System was also obtained [22].

#### Definition of Appropriateness

(An appropriate test is one that is expected to provide more benefit than risk for a patient with a given indication or set of indications).

For all individual examinations, a senior clinical cardiologist independently reviewed clinical and imaging information relating to the request for testing, including review of the patient chart. The first step involved defining the frequency of inappropriate testing. There are currently no specific guidelines or protocols but just some indications regarding when to order a CXR [23]. These last indications were divided into four categories: screening in asymptomatic patients/routine admission, suspected or proven lung pathology, before major surgery, suspected or proven cardiac pathology. Regarding the initial diagnostic assessment of angina the use of CXR is recommended (Class I) in patients with suspected heart failure and in patients with clinical evidence of significant pulmonary disease (ESC guidelines) [24]. The clinical presentation was used to define whether the clinical setting corresponded to an appropriate indication based on the latest specialty guidelines of the American College of Cardiology (ACC)/American Heart Association (AHA) for appropriate use as follows: A  =  definitely appropriate, score 7–9, the procedure should be performed (Class I of Recommendations, Class IIa  =  probably appropriate, it is reasonable to perform the test; benefit > risk), U =  uncertain, score 4–6, the procedure may be considered (Class IIb  =  probably inappropriate, benefit ≥ risk), or I =  definitely inappropriate, score 1–3, the procedure should not be performed (Class III  =  definitely inappropriate, risk > benefit). Indications for CCT were divided into seven categories: symptomatic, asymptomatic, risk assessment without prior test results, risk assessment with prior test results, risk assessment: preoperative evaluation for non-cardiac surgery, post-revascularization (PCI or CABG) according to the latest appropriateness guidelines [25]. Indications for CA were divided into six categories: acute coronary syndrome, without non-invasive stress imaging, with prior non-invasive test results, post-revascularization or myocardial infarction, post-arrhythmias and before major surgery based on the latest (2012) guidelines of the ACCF/AHA for the appropriate use of diagnostic catheterization [26]. Indications for PCI were divided into three categories: acute coronary syndrome, without prior bypass surgery, with prior bypass surgery based on the latest (2012) guidelines of the ACCF/AHA for the appropriate use of coronary revascularization [27]. For each procedure the clinical presentation was allocated within the principal category, and it was graded according to the subcategories/scenarios as being used by the scientific societies and according to patient CAD risk.

The second step involved categorizing the causes of inappropriateness (in classes IIb and III), using groupings according to European Union Medical Imaging guidelines (2001) [28], into one of six possible broad categories:

1. Repeating tests that have already been done (e.g., at another hospital).
2. Investigation when results are unlikely to affect patient management (e.g., because the anticipated positive finding is usually irrelevant or because a positive finding is so unlikely).
3. Investigating too often (e.g., before the disease could have progressed or resolved, or before the results could influence treatment).
4. Do the wrong test.
5. Failing to provide appropriate clinical information and questions that the imaging investigation should answer.
6. Excessive investigation. Some clinicians tend to rely on tests more than others, and some patients have inappropriate expectations of the optimal type of examination.

### Statistical Analysis

Continuous data are expressed as mean ± SD, and dichotomous variables as percentages. We compared continuous data with unpaired-sampled Student's t-test and proportions by X^2^ statistics. We considered statistically significant a *p*-value <.05.

## Results

### Patients

The clinical presentation of the 818 patients is reported in Table 1, and broken down into the type of examination performed. Fifty-five percent of patients underwent two radiation imaging examinations, 24% underwent three. Patients undergoing CCT were younger with a lower risk factor profile (Table 1).

**Table 1:** Patient characteristics.

	*CXR*	*CCT*	*CA*	*PCI*
Number of patients	233	240	245	200
Age (yrs)	67±14	63±11	67±10	67±10
Gender (M/F)	120/117	161/79	185/60	160/40
Diabetes (%)	17	19	18	25
Hypertension (%)	60	27	50	65
Hyperlipidemia (%)	65	34	65	78
Smoking history (%)	65	16	70	51
Prior MI (%)	30	12	37	34
Prior PCI or CABG (%)	15	25	29	37
Chest pain history (%)	51	27	62	60
Dyspnea (%)	24	20	25	20
Rest ECG normal (%)	48	72	11	12

M  =  male; F  =  female; MI  =  myocardial infarction; PCI  =  percutaneous coronary intervention; CABG  =  coronary artery bypass graft; CXR  =  chest x-ray; CCT  =  coronary computed tomography; CA =  diagnostic invasive coronary angiography.

### Appropriateness

**Figure 1:**
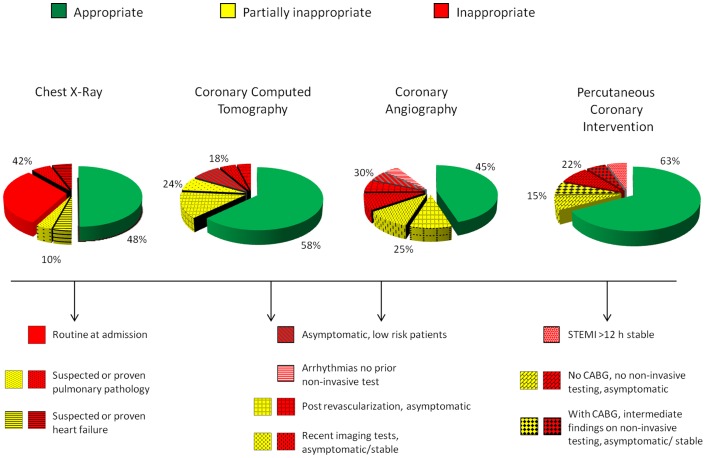
Rate of appropriateness for the four procedures. Pie graph show the rate of appropriate (green slices), partially appropriate (yellow slices) and inappropriate (red slices) examinations for 4 different tests: chest x-ray, coronary computed tomography, diagnostic invasive coronary angiography and coronary percutaneous interventions. The top reasons for inappropriate and partially inappropriate testing are indicated for the four procedures as reported in Table 2. CCT: coronary computed tomography; CA: coronary angiography; PCI: percutaneous coronary intervention.

The top reasons for inappropriateness are listed in Table 2. Screening in asymptomatic patients and/or as routine admission was the top indication for CXR. There were significant differences in the rate of the indications between CCT and CA except for follow-up testing post-revascularization (Table 2). The appropriateness score for each imaging test is shown in Fig. 1. The inappropriateness rate ranged from 18% of CCT to 42% of CXR. If partially appropriate examinations were also included, the inappropriateness rate ranged from 37% of PCI to 55% of CA.

**Table 2:** Top reasons for inappropriate testing for the four procedures.

	*CXR*	*CCT*	*CA*	*PCI*
Screening in asymptomatic patients/routine at admission%	70			
Suspected or proven pulmonary pathology%	13			
Suspected heart failure	13			
Detection of CAD, asymptomatic, low risk patients%		42	17	
Recent imaging tests, asymptomatic/stable symptoms%		23	27	
Post revascularization, asymptomatic%		20	20	
Arrhythmias no prior non-invasive test%			13	
No CABG, one or two vessel CAD without prox LAD, no non-invasive testing, asymptomatic%				36
With prior CABG, all bypass graft patent, intermediate findings on non-invasive testing, asymptomatic/stable%				23
STEMI >12 h from symptom onset, asymptomatic, stable%				23

CAD = coronary artery disease; CABG =  coronary artery bypass graft; LAD =  left anterior descending; STEMI =  ST Elevation Myocardial Infarction; CXR  =  chest x-ray; CCT  =  coronary computed tomography; CA  =  diagnostic invasive coronary angiography. PCI  =  percutaneous coronary intervention.

The top three reasons for inappropriateness (classes IIb and III) were 1) inconclusive, unlikely to alter management, in 50% of all inappropriate tests, 2) tests performed as first-line test (35%) and 3) tests performed as a part of a regular follow-up program (after an acute event or mechanical revascularization) at regular intervals (from the revascularization or previous stress test) in the absence of any change in clinical status (15% of all inappropriate tests).

## Discussion

This study shows a high rate of inappropriateness in medical imaging testing performed in a high-volume tertiary care referral cardiovascular center in Tuscany, Italy.

### Comparison with Previous Studies

The problem of waste and inappropriateness in medical testing is neither new nor restricted to cardiovascular imaging. A recent Thomson Reuters White paper on healthcare waste estimates unwarranted use as a source of 250–325 billion US dollars annual waste in the USA [1]. Examples of this unwarranted use of services include brand name drugs prescribed when generic alternatives are available or a surgical (or interventional) procedure with a patient-preferred medical treatment alternative. Imaging testing is a major source of waste due to unwarranted use, for several reasons. For instance, diagnostic imaging tests are performed to protect against malpractice exposure, or a high-cost diagnostic procedure is used for patients at low risk for the condition, or a diagnostic test is applied in spite of no expected impact on the course of the treatment or for lack of communication and imperfect exchange of information among physicians. As a result, more than 95 million high-tech scans are done each year in the USA, and medical imaging, including CT, MRI and PET scans, has ballooned into a $100 billion a year industry in the United States, with Medicare paying for $14 billion of that [1]. As many as 20% to 50% should never have been performed because their results did not help diagnose ailments or treat patients [1], [28].The reported inappropriateness is relatively high for all imaging techniques around the world [3], [4], [5], [6], [7], [8]. Therefore, we cannot consider surprising the results of the appropriateness audit performed in our Institution “out of the blue”. Nevertheless, some data are worth noting. First, in our public health system there is no direct professional economic benefit for the practitioner, although there is institutional economic benefit, since hospitals are reimbursed by the Regional Government on a “pay-per-volume” basis, without weighing for appropriateness rate. Second, the clinical theatre was the largest cardiovascular center in Tuscany, a region credited with one of the most advanced models of healthcare in Italy, which in turn is ranked very high in the WHO ranking for quality and equity of healthcare [29]. The Region of Tuscany is the co-owner of the Hospital, and the Regional health plan listed “appropriateness” as one of the four key words in the strategic plan 2008–2010 [30]. Finally, the appropriateness issue and radiation responsibility was first raised in our Institute as a key problem of the sustainability of the healthcare system [31], [32]. Unfortunately, moral suasion in absence of audit and action is not an effective way to change time-honoured prescription habits. It is also interesting that the inappropriateness rate was homogeneously distributed in our sample across all imaging testing procedures, regardless of the cost, radiation dose, and invasiveness. The radiation dose of a Multidetector-row CT is around 750 chest x-rays. A PCI dose ranges from 350 to 2,500 CXRs [33]. They are prescribed with an inappropriateness rate similar to that of a simple CXR.

### Study Limitations

We accepted published guidelines as the only possible gold standard against which to assess appropriateness. The approach to defining appropriateness from guidelines is simple, but it is limited since this process does not allow the evaluation of nuances according to the situation of the patient. In addition, most of the guidelines and society recommendations are based on level of evidence C, that is the consensus of the monitoring committee in the absence of a firm evidence base [34].

The setting of the study is a single tertiary care referral center. All of the ordering physicians and imaging lab physicians were salaried staff physicians who had no direct financial incentive for the performance of additional tests and no financial interest in the imaging equipment. The inappropriateness rate would conceivably be higher if a direct financial incentive were present.

This is a retrospective study and, as such, reflects the real world situation as it is. [35].

### Clinical Implications and conclusions

Our data emphasize the need for urgent action to abate the waste and risks inherent in the application of inappropriate testing. The use of noninvasive imaging in appropriately selected patients translates into life and cost savings. On the other hand useless examinations pose an economic burden to society, restrict access to patients in need, carry acute risks without offering commensurate benefit, and do not increase (and possibly reduce) the quality of health care. As recently emphasized by the February, 2010 FDA initiative [14] to reduce unnecessary medical radiation exposure from medical imaging, and by the International Atomic Energy Agency's 2010 [15] 3A's strategy (Awareness, Appropriateness, Audit), every effort should be made by scientific and political authorities to achieve the currently elusive goal of having each patient get the right imaging exam with the right dose at the right time. This will help slow fiscal bleeding due to healthcare waste, prevent avoidable long-term cancer risk due to radiation exposure, and improve the quality of healthcare [35].
